# The impact of COVID-19 continuous containment and mitigation strategy on the epidemic of vector-borne diseases in China

**DOI:** 10.1186/s13071-022-05187-w

**Published:** 2022-03-05

**Authors:** Xiangyu Guo, Chenjin Ma, Lan Wang, Na Zhao, Shelan Liu, Wangli Xu

**Affiliations:** 1 Center for Applied Statistics, School of Statistics, Renmin University of China, Beijing, 100872 China; 2 College of Statistics and Data Science, Faculty of Science, Beijing University of Technology, Beijing, 100124 China; 3 Department of Geriatrics, The First Affiliated Hospital–Zhejiang University School of Medicine, Hangzhou, 310003 China; 4 School of Ecology and Environment, Anhui Normal University, Wuhu, 241002 Anhui Province China; 5 Department of Infectious Diseases, Zhejiang Provincial Center for Disease Control and Prevention, Hangzhou, 310051 Zhejiang Province China

**Keywords:** COVID-19, Vector-borne diseases, Mitigation and contamination strategy, Mortality, Mobility, Prediction

## Abstract

**Background:**

This study explored the effect of a continuous mitigation and containment strategy for coronavirus disease 2019 (COVID-19) on five vector-borne diseases (VBDs) in China from 2020 to 2021.

**Methods:**

Data on VBDs from 2015 to 2021 were obtained from the National Health Commission of the People’s Republic of China, and the actual trend in disease activity in 2020–2021 was compared with that in 2015–2019 using a two-ratio Z-test and two proportional tests. Similarly, the estimated trend in disease activity was compared with the actual trend in disease activity in 2020.

**Results:**

There were 13,456 and 3684 average yearly cases of VBDs in 2015–2019 and 2020, respectively. This represents a decrease in the average yearly incidence of total VBDs of 72.95% in 2020, from 0.9753 per 100,000 population in 2015–2019 to 0.2638 per 100,000 population in 2020 (*t* = 75.17,* P* < 0.001). The observed morbidity rates of the overall VBDs were significantly lower than the predicted rates (47.04% reduction;* t* = 31.72,* P* < 0.001). The greatest decline was found in dengue, with a 77.13% reduction (observed rate vs predicted rate: 0.0574 vs. 0.2510 per 100,000;* t* = 41.42,* P* < 0.001). Similarly, the average yearly mortality rate of total VBDs decreased by 77.60%, from 0.0064 per 100,000 population in 2015–2019 to 0.0014 per 100,000 population in 2020 (*t* = 6.58,* P* < 0.001). A decreasing trend was also seen in the monthly incidence of total VBDs in 2021 compared to 2020 by 43.14% (t = 5.48, P < 0.001).

**Conclusions:**

The results of this study verify that the mobility and mortality rates of VBDs significantly decreased from 2015–2019 to 2020–2021, and that they are possibly associated to the continuous COVID-19 mitigation and contamination strategy implemented in China in 2020–2021.

**Graphical Abstract:**

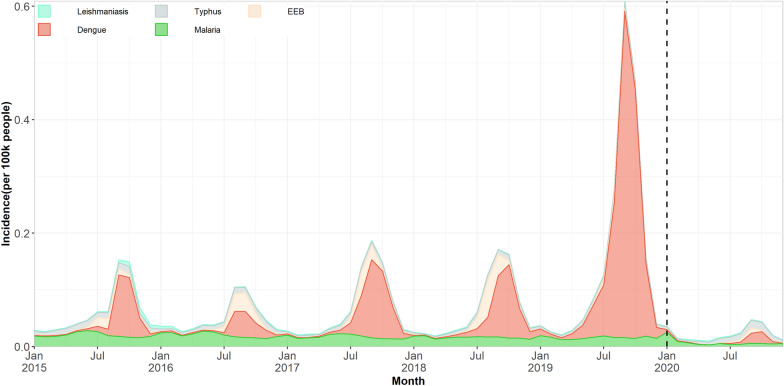

**Supplementary Information:**

The online version contains supplementary material available at 10.1186/s13071-022-05187-w.

## Background

Coronavirus disease 2019 (COVID-19) is a contagious disease caused by severe acute respiratory syndrome coronavirus 2 (SARS-CoV-2), which was first reported in Wuhan, China in 2019 [1]. In January 2020, the WHO Director General declared COVID-19 to be a Public Health Emergency of International Concern [2]. As of 21 January 2022, COVID-19 has affected over 340 million individuals worldwide, resulting in about 5.5 million deaths [3]. China was the first country to be seriously affected by the catastrophic pandemic, and the Chinese national government made great efforts to curb outbreaks of the pandemic. These efforts have included the adoption of many strict measures that have been very effective, even though they have brought great inconvenience to the social and economic lives of the Chinese people. Containment, suppression and mitigation strategies in response to COVID-19 outbreaks, such as the detection and management of cases, lockdowns, prohibition of intercity travel and some protective measures at the individual level [4]. These interventions have achieved great results in China, and because of public health workers’ and clinicians’ diligence, the transmission of COVID-19 has decreased dramatically [5], such that so far China has successfully controlled its COVID-19 epidemic [4]. However, one interesting phenomenon that has become evident is that the incidence and mortality rates of a number of other diseases were lower in the years of strict implementation of COVID-19 measures than in preceding years. The number of outpatient visits involving children with notifiable infectious diseases significantly decreased in the Beijing Chao-Yang Hospital Western Branch during the outbreak of COVID-19 (19 January to 15 April 2020) (influenza, in particular) compared to 2019 [6]. New Zealand’s lower all-cause mortality rate also benefited from strict public health measures during this period [7]. In Taiwan, the reported rate of many infectious diseases, such as severe influenza, dengue and measles, declined in 2020 [8]. Similarly, seasonal influenza had a downward trend in 2020 in Japan [9] and Korea [10]. However, increased deaths attributed to some non-infectious causes also occurred in some areas; ror example, excess deaths occurred in the USA during this period that were not attributable to COVID-19 [11]. Based on these findings, we decided to examine the trend in a number of infectious diseases, to see whether the measures used during the outbreak of COVID-19 played a role in preventing other infectious diseases in China.

Vector-borne diseases (VBDs) are human illnesses caused by parasites, viruses and bacteria that are transmitted by vectors; VBDs include dengue, malaria, epidemic encephalitis B (EEB) and many more. More than 17% all infectious diseases worldwide are VBDs, and they cause over 700,000 deaths each year [12]. From 2008 to 2017, many VBDs steadily decreased among young people in China; for example, the incidence of malaria went from 1.4 per 100,000 population in 2008 to 0.012 per 100,000 population in 2018. In contrast, the incidence of dengue went from 0.009 per 100,000 population in 2008 to 0.19 per 100,000 population in 2018 [13]. In China, there is an outbreak of dengue approximately every 4–6 years, with a peak incidence recorded in 2014. This VBD is prevalent from June to December, and students, service staff and farmers are among the most susceptible groups. VBDs are associated with heavy healthcare and economic burdens and represent serious healthcare challenges to areas all over the world, but especially tropical and subtropical regions. First, these diseases have complicated infectious and epidemic processes involving humans, animals and vectors [14]. Second, patients may suffer a number of VBDs simultaneously. Third, patients with VBDs could have similar symptoms that might be difficult for doctors to diagnose efficiently [15]. A firm control of VBDs cannot be achieved without effective and complete surveillance systems, so many countries have established surveillance and response systems, such as European countries [16], the USA [17] and China [18]. Because climate and human activities affect the habitats and behaviors of the vectors, the occurrence of VBDs is sensitive to climate and change in people’s habits [19].

During the outbreak of COVID-19, measures and interventions greatly changed the daily life of the general population and affected many disease activities, such as gastrointestinal and respiratory infectious diseases [8]. The aim of this study was to determine whether the interventions and measures introduced to manage COVID-19 really work to reduce the incidence and mortality rates of VBDs in Mainland China. To our knowledge, no similar studies have been carried out in China to date.

## Methods

### Strategies for COVID-19 in China

COVID-19 was first reported in Wuhan, China in late December 2019 [1]. In the early stage of the COVID-19 outbreak, the national government adopted a containment strategy in high-risk areas that aimed to block virus transmission. Consequently, each provinces developed surveillance and response systems. In addition, most cities implemented lockdowns, travel bans and school closures. On the personal level, the public was instructed to avoid (large) gatherings and stay at home as much as possible. People were required to wear masks and ensure that they were sufficiently protected if they had to go out. A suppression strategy was then followed during which there was improvement in preventing COVID-19. Although all measures continued to be in effect, the level depended on local surveillance and risk rates; for example, the lockdown or travel ban was less strict. However, the general population was still required to have dependable self-protection. Mitigation was another strategy that was implemented; as this strategy is much less strict than suppression, its implementation depends on the risk level of COVID-19. With mitigation measures, social distancing gradually returned to normal, and harsh measures, such as lockdowns or travel prohibitions, were canceled [20–22].

The Joint Prevention and Control Mechanism of the State Council of China divided 2020 into two phases: the emergency stage and the routine stage. In the emergency stage (January to April 2020), all provinces gradually declared a Level 1 response to public health emergencies; during this period, the corresponding strategy was containment in which different stringent measures were implemented. In the routine stage (May to December 2020), most provinces adopted Level 2 or the lower-level response to public health emergencies, with the exception of partial high-risk areas; in this phase, the corresponding strategies were suppression and mitigation, and stress was put on prevention. The dates of the implementation of the responses to this public health emergency by each province are shown in Additional file 1: Table S1.

### Infectious Diseases’ Information Report System in China

After going through the baptism of SARS, China constructed a direct Internet reporting system for infectious diseases in 2004 that collects information on reports of infectious diseases, case management, basic public health data, special disease reports and statistical analysis [23, 24]. In this system, 40 notifiable diseases (including 6 VBDs: EEB, typhus, dengue, malaria, leishmaniasis and filariasis; see Additional file 1: Table S2) are covered and reported within a stipulated time, depending on the risk class of the corresponding infectious diseases (2 class A diseases, 27 class B diseases and 11 class C diseases). A very clear explanation of the reporting system can be found in the standardized regulation of infectious disease information reports by the National Health Commission of the People’s Republic of China [24].

### Data collection

Data on VBDs for the period 2015–2020 and for January to April 2021 were obtained from the National Health Commission of the People’s Republic of China, from which we could download relative notifiable disease data from the Chinese Mainland; this Commission updates the number of cases and deaths monthly. In this study, we extracted all VBDs from the data on notifiable diseases by their transmission routes (vector-borne), including EEB, dengue, malaria, filariasis, typhus and leishmaniasis. The details of these diseases are listed in Additional file 1: Table S2. Since the cases and mortalities of filariasis in 2015–2020 were zero, we excluded this disease from any subsequent analysis. The population data were obtained from the National Bureau of Statistics of the People’s Republic of China and are updated at the end of every year.

### Statistical analysis

Using the basic VBD data obtained from the National Health Commission of the People’s Republic of China, we determined the monthly and yearly average incidence and mortality rates of each disease. The relative changes in the rates were also calculated.

To infer whether interventions implemented for COVID-19 affected the incidence of VBDs, we carried out a series of statistical procedures. We used the two proportional tests and two-ratio Z-tests to test the difference in relative rates between 2020 and the previous 5 years. We also used Farrington surveillance algorithms [25] to test the long-term downward trend in each disease.

We also predicted the corresponding rates of VBDs based on the data from 2015–2019. Hence, we could observe the trend in VBDs, based on the supposition that COVID-19 did not surface and no measures were taken. We thought we might find something interesting if we compared the trend with the real trend; therefore, for each VBD, the estimated monthly cases and mortalities from January to December 2020 were generated using Farrington surveillance algorithms [8, 25] based on the corresponding values from the period 2015–2019. In addition, a 95% confidence interval (CI) was estimated using Farrington surveillance algorithms (Farrington surveillance algorithms can be used with the surveillance package in R; the relative tests and plot were also implemented in R; R Foundation for Statistical Computing, Vienna, Austria).

## Results

### Overall trend in yearly incidence of VBDs

A total of 70,965 cases of VBDs were reported from Mainland China in the period 2015–2020 (an average of 11,828 cases annually). The average yearly incidence of total VBDs decreased by 72.95% between 2015–2019 and 2020, from 0.9753 per 100,000 population in 2015–2019 to 0.2638 per 100,000 population in 2020 (*t* = 75.17,* P* < 0.001; Table 1). A dramatic decrease in disease activity was seen in four of the VBDs studied in 2020 (all* P* < 0.01), with the greatest decline seen in dengue (89.93%;* t* = 76.09,* P* < 0.001) (Table 1). In contrast, no changes were seen in the incidence of typhus, which showed a 1.01% escalation (*t* = 0.24,* P* < 0.811). Exploring these data in greater detail, it became evident that the activities of the vaccine-preventable and viral VBDs decreased to a much greater extent than those of the non-vaccine-preventable and non-viral VBDs (all* P* < 0.001; Additional file 1: Table S3).

**Table 1:** Changes in the average annual incidence of five vector-borne diseases in 2020 compared to the previous 5 years 2015–2019) in China

Diseases	Average yearly incidence		Average yearly cases (*n*)		Percentage change^a^ (95% CI)	*P*-value^b^
	2020	2015–2019	2020	2015–2019		
Overall	0.2638	0.9753	3684	13,456	− 72.95 (− 74.85 to − 71.05)	< 0.001
EEB	0.0223	0.0844	312	1165	− 73.53 (− 79.99 to − 67.09)	< 0.001
Typhus^c^	0.0859	0.0850	1199	1173	1.01 (− 7.10 to 9.08)	0.81
Dengue	0.0574	0.5704	802	7870	− 89.93 (− 92.25 to − 87.62)	< 0.001
Malaria^c^	0.0816	0.2147	1140	2962	− 61.98 (− 66.20 to − 57.75)	< 0.001
Leishmaniasis^c^	0.0165	0.0208	231	287	− 20.48 (− 35.94 to − 5.02)	0.01

CI, Confidence interval; EEB epidemic encephalitis B

^a^Changes = (x1 − ×2)/x2 × 100%, where x1 is the average yearly incidence in 2020, and x2 is the average yearly incidence in the previous 5 years (2015–2019)

^b^The* P*-value was computed using two proportional tests

^c^The monthly incidence has a long-term significant downward trend

### Overall trend in monthly incidence of VBDs

**Fig. 1:**
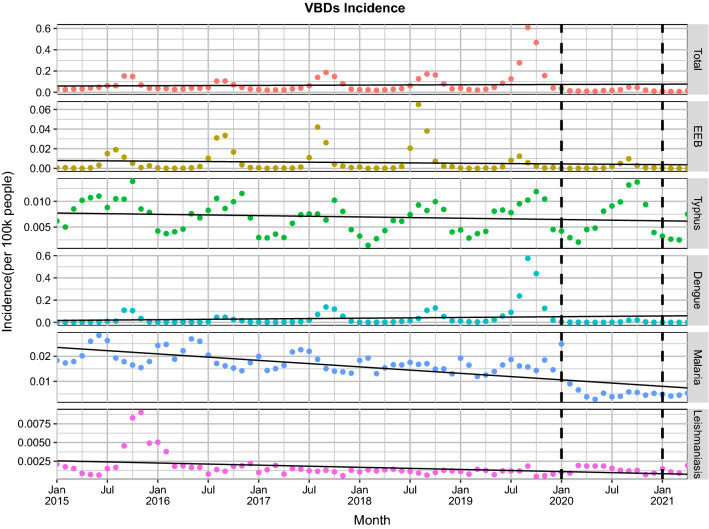
Monthly incidence rates and trends of 5 vector-borne diseases from January 2020 to April 2021 compared with the 5 preceding years (2015–2019) in China. The linear trend is fitted by the linear regression.* Abbreviations*: EEB, Epidemic encephalitis B

**Fig. 2:**
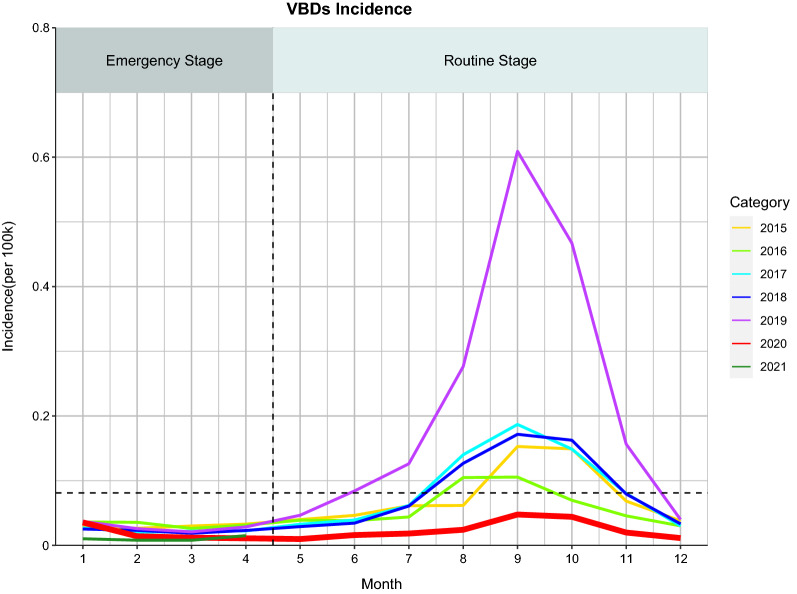
Comparison of monthly incidence rates of 5 vector-borne diseases during 2015–2019 and 2020 and 2021 in China (stratifying incidence by years). The horizontal dashed line represents the average of the monthly incidence in 2015–2019 (the average of 60 monthly incidences over the previous 5 years)

A decreasing trend was also seen in the monthly incidence of four VBDs in 2020–2021 compared to the previous years (Fig. 1). The monthly incidence in the overall VBDs was significantly lower in 2020 and 2021 compared with that in the same period of 2015, 2016, 2017, 2018 and 2019 (Fig. 2).

Further analysis by pandemic stage showed that the overall incidence rates for all VBDs decreased significantly by 33.04% (*t* = 4.95, *P* < 0.001) during the emergency (January–April 2020) compared to the same period in the previous 5 years and further decreased by 43.14% (*t* = 5.48,*P* < 0.001) in the same period of 2021 (Additional file 1: Table S4).

### Overall trend in yearly morality due to VBDs

There were 461 deaths from all the VBDs reported from Mainland China in 2015–2020. The average yearly mortality rate of total VBDs was 0.0014 per 100,000 population in 2020 and 0.0064 per 100,000 population in the period 2015–2019, which represents a decrease of 77.60% (*t* = 6.58, *P* < 0.001; Table 2) in 2020. The mortality rates from EEB decreased by 83.67% in 2020, from 0.0053 per 100,000 population in 2015–2019 to 0.0009 per 100,000 population in 2020 (*t* = 6.64, *P* < 0.001; Table 2). However, the mortality rates of the other three VBDs studied (typhus, dengue and leishmaniasis) were almost 0 (Table 2). The mortality rates of the vaccine-preventable and viral VBDs decreased significantly faster than those of the non-vaccine-preventable and non-viral VBDs (Additional file 1: Table S5).

**Table 2:** Changes in average yearly mortality due to five vector-borne diseases in 2020 compared to the previous 5 years (2015–2019) in China

Diseases	Average yearly mortality rates		Average yearly deaths (*n*)		Percentage change^a^ (95% CI)	*P*-value^b^
	2020	2015–2019	2020	2015–2019		
Overall	0.0014	0.0064	20	88	− 77.60 (− 100.64 to − 54.45)	< 0.001
EEB	0.0009	0.0053	12	73	− 83.67 (− 108.47 to − 59.05)	< 0.001
Typhus	0.0000	0.0000	0	0	NA	NA
Dengue	0.0000	0.0001	0	1	− 100.00 (− 296.00 to 96.00)	0.32
Malaria	0.0005	0.0010	7	14	− 49.15 (− 114.50 to 13.30)	0.12
Leishmaniasis	0.0001	0.0000	1	0	NA	NA

^a^Changes = (x1 − ×2)/x2 × 100%, where x1 is the average yearly incidence in 2020, and x2 is the average yearly incidence in the previous 5 years (2015–2019)

^b^The* P*-value was computed using two proportional tests

### Overall trend in monthly mortality due to VBDs

**Fig. 3:**
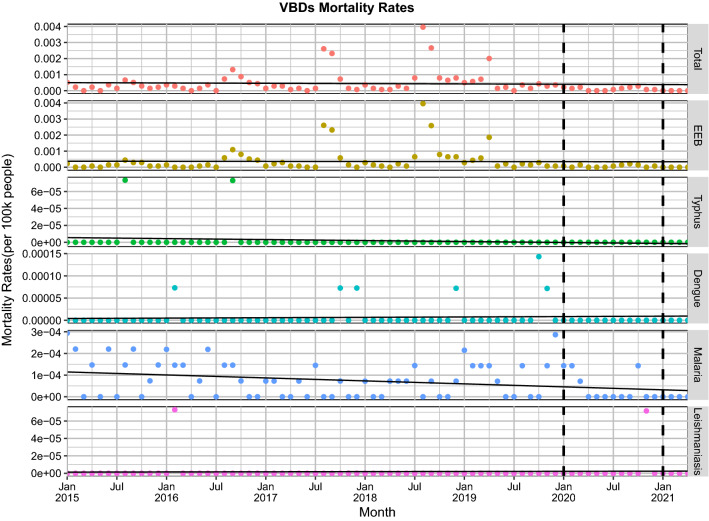
Monthly mortality rates and trends of 5 vector-borne diseases from January 2020 to April 2021 compared with the five preceding years (2015–2019) in China. The linear trend is fitted by the linear regression

**Fig. 4:**
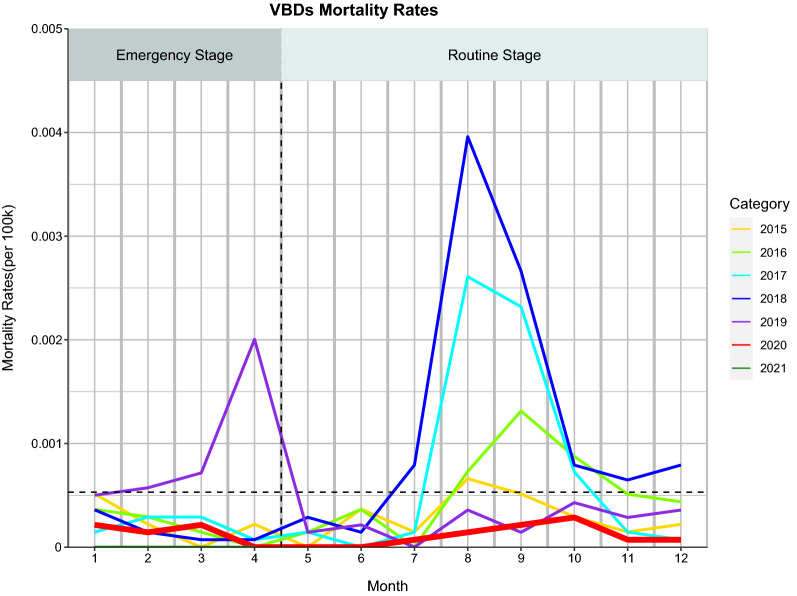
Comparison of monthly mortality rates of 5 vector-borne diseases during 2015–2019 and 2020 and 2021 in China (stratifying incidence by years). The horizontal dashed line represents the average of monthly mortality rates in 2015–2019 (the average of 60 monthly incidences over the previous 5 years)

A decreasing trend was found in the mean monthly mortality rate in each VBD between the same period of 2020–2021 and 2015–2019 (Fig. 3). The monthly mortality rate in overall VBDs in 2020–2021 compared to each year in the period 2015–2019 decreased (Fig. 4).

During the 2020 emergency (January to April), the average monthly mortality rate due to overall VBDs was lower than in the same period in 2015 and 2019 (a 59.26% reduction;* t* = 1.10, *P* < 0.27) and in 2021 (a 100% reduction;* t* = 1.41, *P* < 0.15); see Additional file 1: Table S6.

### Comparison of the observed and predicted incidence and mortality of VBDs

The yearly incidence and mortality rates of overall VBDs showed a significant decline, possibly due to two factors: (i) the inherent long-term downward trend in VBDs induced this decline; and (ii) a real influence of the interventions and measures for COVID-19 on VBDs. Based on these two possibilities, we used Farrington surveillance algorithms to verify whether these diseases actually did undergo a long-term downward trend. Analysis with Farrington surveillance algorithms showed that the monthly incidence of typhus, malaria and leishmaniasis had a significant long-term downward trend (Fig. 1; *P* < 0.001). However, the downward trend of typhus was very small compared to the corresponding trend of malaria and leishmaniasis.

**Fig. 5:**
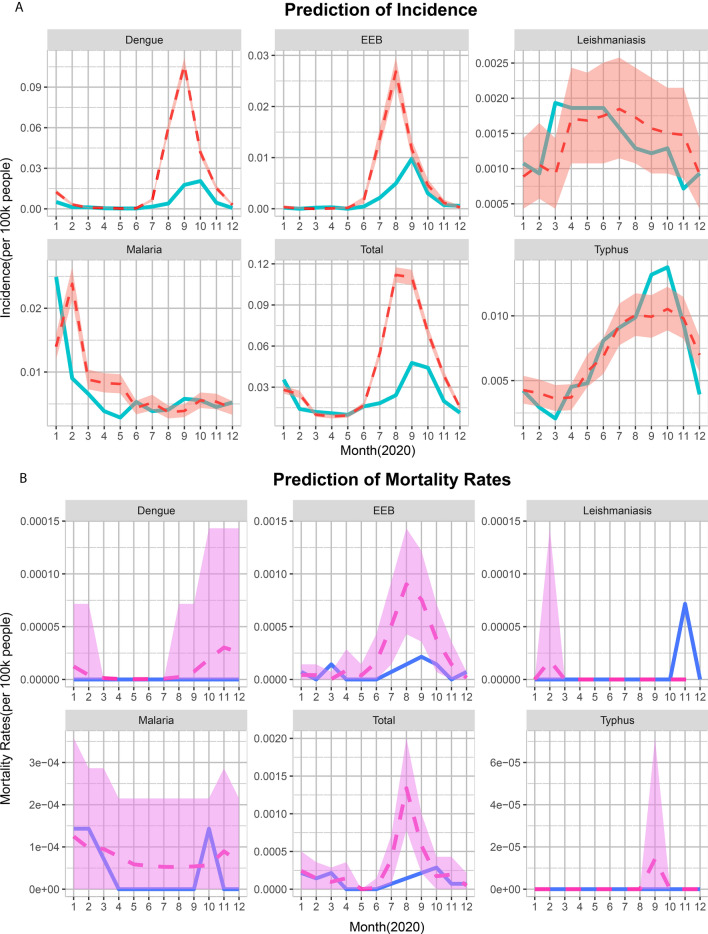
Prediction of the monthly incidence (**a**) and monthly mortality (**b**) rates of vector-borne diseases in 2020 in China. The red dashed lines are the predicted means, the light-blue lines are the observed means and the light-shaded areas are 95% confidence intervals. The predicted monthly cases and mortalities from January to December 2020 were generated using Farrington surveillance algorithms based on the corresponding values from 2015 to 2019

For yearly incidence, a significant decrease in real value was seen in overall VBDs, EEB, dengue and malaria in 2020 (all *P* < 0.01) relative to the predicted value, with the greatest reduction seen for dengue (77.13%;* t* = 41.42, *P* < 0.001 ; Additional file 1: Table S7). Regarding the monthly incidence rate of total VBDs, the real incidences of total VBDs, EEB, dengue and malaria were much lower than the predicted ones, while the observed incidences of typhus and leishmaniasis were similar to the predicted values (Fig. 5a). In the emergency stage of the pandemic (January to April 2020), the observed average monthly incidences of dengue (54.20% reduction) and malaria (19.15% reduction) were significantly lower than the predicted ones (all *P*-values < 0.05). In the routine stage (May to December 2020), the observed average monthly incidences of the diseases overall (55.18% decreased), dengue (78.81% decreased) and EEB (64.01% decreased) were lower than the predicted ones as well (all *P*-values < 0.001). For more details, see Additional file 1: Table S8.

For the corresponding mortality rates, the real value of the overall VBDs decreased by 61.89% more than the predicted points (Additional file 1: Table S9). However, the predicted monthly mortality rates of the VBDs were nearly identical to the actual values (Fig. 5b). The same result was seen in the observed and predicted values during the emergency and routine stages of the pandemic (Additional file 1: Table S10).

## Discussion

Using nationally representative mobility and mortality data for VBDs before, during and after the outbreak of COVID-19 in 31 provinces of China, the results of this study show that during the 2020 COVID-19 outbreak, the average yearly incidence and mortality rates of VBDs in Mainland China declined notably, with a decrease of 72.95% and 77.60%, respectively. Dengue accounted for 56.14% of the overall VBDs, and its incidence and mortality rates showed the greatest decrease among all of the VBDs assessed. Likewise, the observed incidence and mortality rates of the overall VBDs were much lower than the predicted rates. To our knowledge, this is the first study to assess the influence of the COVID-19 pandemic on the incidence and mortality of VBDs in China, and one with the largest number of samples.

VBDs, such as malaria, dengue and leishmaniasis, exert a huge burden of morbidity and mortality worldwide, and they particularly affect the poorest of the poor [26].

In China, dengue and malaria are the main VBDs, and most cases are imported from other countries. During the period 2013–2016, of all 16,206 imported cases of VBDs in China, 83.12% (13,471) were malaria cases, followed by dengue fever (2628 cases; 16.22%). Both Malaria and dengue cases were imported from South Africa, Asia and South America [27–29]. Consistent with previous reports in Guangzhou and Jiangsu in China and elsewhere in the world [30], the incidence of overall VBDs was significantly lower in 2020 than in 2015–2019. Similarly, the observed monthly incidence of overall and cause-specific VBDs during the 2020 COVID-19 outbreak was lower than the predicted rates. The decreased activity of such diseases may have been affected by the implementation of public health control measures against COVID-19 [4]. Several factors could have contributed to the decreased incidence of malaria and dengue. First, there was a reduction in the number of international travelers during 2020. Second, in 2020, the requirement that international travelers stay in a designated hotel for a 14-day quarantine period faciliated the identification of all imported malaria and dengue cases and further curbed the secondary spread of these diseases in domestic areas [30]. Third, environmental management programs tailored to the ecology and behavior of local vector species, especially* Aedes* and* Anopheles* mosquitoes, were implemented; these contributed to the control of malaria and dengue as these mosquitoes are the most common vectors that transmit several VBDs. Fourth, the chance of being bitten by * Aedes albopictus* mosquitoes was reduced by non-pharmaceutical interventions (NPIs) [30]. Fifth, beginning in 2010 the Chinese government implemented a strategy of malaria eradication (2010–2020) [31]. In subsequent years, indigenous cases of malaria decreased significantly [32, 33]. These measures were also implemented further in the regularization phase of the pandemic. Finally, it is possible that people made fewer visits to clinics, resulting in a lower diagnosis for VBDs, because of the strict quarantine measures and, consequently, a decrease in health-seeking behaviors.

Worldwide, over 1 billion cases of VBD occur annually, causing over 1 million deaths [34]. However, only 108 deaths from VBDs were reported in China during the 2015–2020 period, which is a remarkable decline in the overall mortality rate from VBDs in 2020 compared to the previous 5 years in China. In particular, the number of yearly deaths from EEB and malaria decreased dramatically in 2020. We also observed that the observed monthly mortality rates of overall and specific VBDs in the 2020 COVID-19 pandemic were much lower than the predicted rates. The observed decreased activity of such diseases correlates with several factors. First, all interventions and measures for the COVID-19 pandemic were aimed at greatly reducing the occurrence of VBDs and decreasing mortality. Second, VBDs have flu-like symptoms similar to those of COVID-19 in the early stages, which contributed to them being diagnosed through improved surveillance, testing and diagnosis for COVID-19. Third, the reduction might be due to the difficulty in accessing hospital services or the unwillingness of people to seek hospital care during the outbreak. This is consistent with the findings of a previous study showing that there were significant reductions in hospital deaths [35].

There are several limitations to our study. First, the analyzed data focused on the national level and provided little information on each province. Second, data at the individual level were too limited to enable more detailed analyses by age, gender and other basic disease histories and personal behaviors. The number of VBD cases and deaths due to VBDs during the 2020 COVID-19 pandemic could still be an underestimation, especially in Hubei Province, China.

## Conclusions

The nationwide data assessed in this study show that the incidence of the overall VBDs and the associated mortality rates during the initial outbreak of COVID-19 were reduced by 72.95% and 77.60%, respectively, compared to the previous 5 years, with the greatest decline being dengue and EEB. We also found that the observed values of incidence and mortality rates were lower than the predicted values in 2020. All of measures reported here for preventing COVID-19 contributed to the decreased incidence of VBDs and the associated mortality in China. Because most VBDs are to non-vaccine-predictable diseases, it will be a major challenge to stop the transmission of VBDs in China in the future. All of the prevention and control strategies for VBDs reported here were similar to those implemented in preceding years, including improving vector and disease surveillance and environmental management for public health workers, developing rapid diagnosis kits, issuing public education messages, preventing further vector exposure in patients, cleaning up vector breeding sites and using pesticides. Rapid and coordinated responses to contain, suppress and eradicate the local transmission of VBDs is vital because such responses can minimize detrimental effects on public health, medical facilities and societal and economic activities.

## Supplementary Information

**Additional file 1: Table S1.** Dates of provinces implementing response to public health emergency. **Table S2. **List of five vector-borne diseases, insect vectors, and pathogens. **Table S3.** Changes in the average yearly incidence of vaccine-preventable, non-vaccine-preventable, viral, and non-viral diseases among five vector-borne diseases in 2020 were compared with the previous five years in China. **Table S4.** Pairwise comparisons of the average monthly incidence of five vector-borne diseases for January to April in 2015–2019, 2020 and 2021. **Table S5**. Changes in the average yearly mortality rates of the vaccine-preventable, non-vaccine-preventable, viral, and non-viral diseases among five vector-borne diseases in 2020 compared with the previous five years in China. **Table S6.** Pairwise comparisons of the average monthly mortality rates of five vector-borne diseases for January to April in 2015–2019, 2020 and 2021. **Table S7.** Changes in the yearly incidence of five vector-borne diseases in 2020 between observed and predicted values. **Table S8.** Changes in the average monthly incidence of five vector-borne diseases in the emergency response stage (January to April 2020) and the routine response stage (May to December 2020) between observed and predicted values. **Table S9.** Changes in the yearly mortality rates of five vector-borne diseases in 2020 between observed and predicted values. **Table S10.** Changes in the average monthly mortality rates of five vector-borne diseases in the emergency response stage (January to April 2020) and the routine response stage (May to December 2020) between observed and predicted values.

## Data Availability

Notifiable disease data from China can be obtained from the website of the National Health Commission of the People’s Republic of China. (For example: the notifiable disease data from China for October 2020 can be downloaded from http://www.nhc.gov.cn/cms-search/xxgk/getManuscriptXxgk.htm?id=e6af1cf101bb4ebeb84ef2dd65d3f68a)
