# Supplementary material for: The impact of COVID-19 continuous containment and mitigation strategy on the epidemic of vector-borne diseases in China
Source: Parasit Vectors. 2022 Mar 5;15:78. doi: 10.1186/s13071-022-05187-w (PMC8898061; doi:10.1186/s13071-022-05187-w)
Supplement: Supplementary file 1 — Additional file 1: Table S1. Dates of provinces implementing response to public health emergency. Table S2. List of five vector-borne diseases, insect vectors, and pathogens. Table S3. Changes in the average yearly incidence of vaccine-preventable, non-vaccine-preventable, viral, and non-viral diseases among five vector-borne diseases in 2020 were compared with the previous five years in China. Table S4. Pairwise comparisons of the average monthly incidence of five vector-borne diseases for January to April in 2015–2019, 2020 and 2021. Table S5. Changes in the average yearly mortality rates of the vaccine-preventable, non-vaccine-preventable, viral, and non-viral diseases among five vector-borne diseases in 2020 compared with the previous five years in China. Table S6. Pairwise comparisons of the average monthly mortality rates of five vector-borne diseases for January to April in 2015–2019, 2020 and 2021. Table S7. Changes in the yearly incidence of five vector-borne diseases in 2020 between observed and predicted values. Table S8. Changes in the average monthly incidence of five vector-borne diseases in the emergency response stage (January to April 2020) and the routine response stage (May to December 2020) between observed and predicted values. Table S9. Changes in the yearly mortality rates of five vector-borne diseases in 2020 between observed and predicted values. Table S10. Changes in the average monthly mortality rates of five vector-borne diseases in the emergency response stage (January to April 2020) and the routine response stage (May to December 2020) between observed and predicted values. [file 13071_2022_5187_MOESM1_ESM.docx]

**Additional files**

**Table S1. Dates of provinces implementing response to public health emergency**

| **Province** | **Public health emergency response level 1** | **Public health emergency response level 2** | **Public health emergency response level 3** | **Public health emergency response level 4** |
| --- | --- | --- | --- | --- |
| **Beijing** | January 24 | April 30 |  |  |
| **Tianjin** | January 24 | April 30 |  |  |
| **Hebei** | January 24 | April 30 |  |  |
| **Shanxi** | January 25 | February 24 | March 10 |  |
| **Inner-Mongolia** | January 25 |  | February 25 |  |
| **Liaoning** | January 25 |  | February 22 |  |
| **Jilin** | January 25 | February 26 | March 20 |  |
| **Heilongjiang** | January 25 | March 4 | March 25 |  |
| **Shanghai** | January 24 | March 24 |  |  |
| **Jiangsu** | January 24 | February 24 | March 27 |  |
| **Zhejiang** | January 23 | March 2 | March 23 |  |
| **Anhui** | January 24 | February 25 | March15 |  |
| **Fujian** | January 24 | February 26 | February 26 |  |
| **Jiangxi** | January 24 | March 12 | March 20 |  |
| **Shandong** | January 24 | March 7 | May 5 |  |
| **Henan** | January 25 | March 19 | May 5 |  |
| **Hubei** | January 24 | May 2 |  |  |
| **Hunan** | January 23 | March 10 | March 31 |  |
| **Guangdong** | January 23 | February 24 |  |  |
| **Guangxi** | January 24 |  | February 24 |  |
| **Hainan** | January 25 | January 24 | February 26 |  |
| **Chongqing** | January 24 | March 10 | March 24 |  |
| **Sichuan** | January 24 | February 26 | March 25 |  |
| **Guizhou** | January 24 |  | February 23 |  |
| **Yunnan** | January 24 |  | February 24 |  |
| **Xizang** | January 30 | March 6 |  |  |
| **Shaanxi** | January 25 |  | February 28 |  |
| **Gansu** | January 25 |  | February 21 |  |
| **Qinghai** | January 25 |  | February 26 | March 6 |
| **Ningxia** | January 25 | February 28 |  |  |
| **Xinjiang** | January 25 | February 25 | March 7 | March 21 |

**Table S2. List of five vector-borne diseases, insect vectors, and pathogens**

| **Vector** | **Diseases** | **Pathogen** | **Classification** |
| --- | --- | --- | --- |
| Mosquito | EEB | Virus | B |
| Mosquito | Dengue Fever | Virus | B |
| Mosquito | Malaria | Parasite | B |
| Mosquito | Filariasis | Parasite | C |
| Lice | Typhus | Bacteria | C |
| Sandflies | Leishmaniasis | Parasite | C |

**Note:** There were six vector-borne diseases, the corresponding insect vector and pathogen was given. Data from Chinese Law on the Prevention and Control of Infectious Diseases in the Republic of China issued on Aug 30, 2018, released by National Health Commission of the People’s Republic of China, Cited from

http://www.nhc.gov.cn/fzs/s3576/201808/6d00c158844f42c5bcf94993bffa665a.shtml

**Table S3. Changes in the average yearly incidence of vaccine-preventable, non-vaccine-preventable, viral, and non-viral diseases among five vector-borne diseases in 2020 were compared with the previous five years in China**

| **Disease classification** | **Average yearly incidence** | | **Changes (%)**  **95%CI** | **P value** |
| --- | --- | --- | --- | --- |
|  | **2020** | **2015-2019** |  |  |
| **Vaccine-preventable diseases** | 0.0223 | 0.0844 | -73.53 (-79.98 to -67.08) | <0.001 |
| **Non-vaccine-preventable diseases** | 0.2415 | 0.8909 | -72.90 (-74.88 to -70.91) | <0.001 |
| **Viral diseases** | 0.0798 | 0.6548 | -87.82 (-89.99 to -85.63) | <0.001 |
| **Non-viral diseases*** | 0.1840 | 0.3205 | -42.58 (-46.27 to -38.89) | <0.001 |

**Note:** Changes = (x1-x2)/x2×100%; x1: average yearly incidence in 2020; x2: average yearly incidence in the previous five years (2015–2019); the p-value was computed through two proportional tests. The superscripts * means that the monthly incidence of this disease has a long-term significant downward trend.

**Table S4. Pairwise comparisons of** **the average monthly incidence of five vector-borne diseases for** **January to April in 2015–2019, 2020 and 2021**

| **disease** | **2015-2019** | **2020** | **2021** | **2015–2019 vs. 2020** | | **2015–2019 vs. 2021** | | **2020 vs. 2021** | |
| --- | --- | --- | --- | --- | --- | --- | --- | --- | --- |
|  |  |  |  | **Change (%)** | **P value** | **Change (%)** | **P value** | **Change (%)** | **P value** |
| **Total** | 0.0271 | 0.0181 | 0.0103 | -33.04 | <0.001 | -61.92 | <0.001 | -43.14 | <0.001 |
| **EEB** | 0.0004 | 0.0002 | 0.0001 | -52.95 | 0.31 | -81.18 | 0.08 | -60.00 | 0.42 |
| **Typhus** | 0.0043 | 0.0034 | 0.0040 | -19.76 | 0.26 | -7.22 | 0.68 | 15.63 | 0.46 |
| **Dengue** | 0.0030 | 0.0020 | 0.0002 | -33.49 | 0.09 | -93.35 | <0.001 | -90.00 | <0.001 |
| **Malaria** | 0.0178 | 0.0111 | 0.0047 | -37.56 | <0.001 | -73.51 | <0.001 | -57.58 | <0.001 |
| **Leishmaniasis** | 0.0017 | 0.0015 | 0.0014 | -13.76 | 0.62 | -19.09 | 0.49 | -6.17 | 0.84 |

**Note:** Changes = (x1-x2)/x2 × 100%; x1: average monthly incidence (mortality rates) in 2020; x2: average monthly incidence (mortality rates) in the previous five years (2015–2019); the p-value was computed through two proportional tests.

**Table S5. Changes in the average yearly mortality rates of the vaccine-preventable, non-vaccine-preventable, viral, and non-viral diseases among five vector-borne diseases in 2020 compared with the previous five years in China**

| **Disease classification** | **Average yearly mortality rates** | | **Changes (%)**  **95%CI** | **P value** |
| --- | --- | --- | --- | --- |
|  | **2020** | **2020** |  |  |
| **Vaccine-preventable diseases** | 0.0009 | 0.0053 | -83.67 (-108.47 to -59.04) | <0.001 |
| **Non-vaccine-preventable diseases** | 0.0006 | 0.0011 | -49.33 (-110.37 to 9.17) | 0.09 |
| **Viral diseases** | 0.0009 | 0.0054 | -83.98 (-108.50 to -59.45) | <0.001 |
| **Non-viral diseases** | 0.0006 | 0.0010 | -44.34 (-108.92 to 21.83) | 0.19 |

**Note:** Changes = (x1-x2)/x2 × 100%; x1: average yearly mortality rates in 2020; x2: average yearly mortality rates in the previous five years (2015–2019); the p-value was computed through two proportional tests.

**Table 6. Pairwise comparisons of the average monthly mortality rates of five vector-borne diseases for January to April in 2015–2019, 2020 and 2021**

| **disease** | **2015–2019** | **2020** | **2021** | **2015–2019 vs. 2020** | | **2015–2019 vs. 2021** | | **2020 vs. 2021** | |
| --- | --- | --- | --- | --- | --- | --- | --- | --- | --- |
|  |  |  |  | **Change (%)** | **P value** | **Change (%)** | **P value** | **Change (%)** | **P value** |
| **Total** | 0.0004 | 0.0001 | 0.0000 | -59.26 | 0.27 | -100.00 | 0.03 | -100.00 | 0.15 |
| **EEB** | 0.0002 | 0.0001 | 0.0000 | -77.55 | 0.2 | -100.00 | 0.07 | -100.00 | 0.38 |
| **Typhus** | 0.0000 | 0.0000 | 0.0000 | NA | NA | NA | NA | NA | NA |
| **Dengue** | 0.0000 | 0.0000 | 0.0000 | -100.00 | 0.82 | -100.00 | 0.82 | NA | NA |
| **Malaria** | 0.0001 | 0.0001 | 0.0000 | -14.83 | 0.89 | -100.00 | 0.22 | -100.00 | 0.26 |
| **Leishmaniasis** | 0.0000 | 0.0000 | 0.0000 | -100.00 | 0.82 | -100.00 | 0.82 | NA | NA |

**Note:** Changes = (x1-x2)/x2 × 100%; x1: average monthly mortality rates in 2020; x2: average monthly mortality rates in the previous five years (2015–2019); the p-value was computed through two proportional tests.

**Table S7. Changes in the yearly incidence of five vector-borne diseases in 2020 between observed and predicted values**

| **Diseases** | **Incidence** | | **Cases** | | **Changes (%)** | **P value** |
| --- | --- | --- | --- | --- | --- | --- |
|  | **Observed** | **Predicted** | **Observed** | **Predicted** |  |  |
| **Total** | 0.2638 | 0.4981 | 3684 | 6956 | -47.04 | <0.001 |
| **EEB** | 0.0223 | 0.0606 | 312 | 846 | -63.19 | <0.001 |
| **Typhus** | 0.0859 | 0.0848 | 1199 | 1184 | 1.30 | 0.75 |
| **Dengue** | 0.0574 | 0.2510 | 802 | 3505 | -77.13 | <0.001 |
| **Malaria** | 0.0816 | 0.0955 | 1140 | 1333 | -14.52 | <0.001 |
| **Leishmaniasis** | 0.0165 | 0.0170 | 231 | 238 | -3.21 | 0.74 |

**Note:** Changes = (x1-x2)/x2 × 100%; x1: yearly incidence (observed) in 2020; x2: yearly incidence (predicted) in 2020; the p-value was computed through two proportional tests.

**Table S8. Changes in** **the average monthly incidence of five vector-borne diseases in the emergency response stage (January to April 2020) and the routine response stage (May to December 2020) between observed and predicted values**

| **Diseases** | **Emergency stage (January to April)** | | | | **Routine stage (May to December)** | | | | **E vs R P-value** |
| --- | --- | --- | --- | --- | --- | --- | --- | --- | --- |
|  | **Observed** | **Predicted** | **Changes (%)** | **P value** | **Observed** | **Predicted** | **Changes (%)** | **P value** |  |
| **Total** | 0.0181 | 0.0178 | 1.61 | 0.85 | 0.0239 | 0.0533 | -55.18 | <0.001 | <0.001 |
| **EEB** | 0.0002 | 0.0001 | 47.09 | 0.69 | 0.0027 | 0.0075 | -64.01 | <0.001 | 0.35 |
| **Typhus** | 0.0034 | 0.0039 | -11.85 | 0.52 | 0.0090 | 0.0087 | 4.20 | 0.74 | 0.47 |
| **Dengue** | 0.0020 | 0.0043 | -54.20 | <0.001 | 0.0062 | 0.0292 | -78.81 | <0.001 | 0.13 |
| **Malaria** | 0.0111 | 0.0137 | -19.15 | 0.04 | 0.0047 | 0.0051 | -8.18 | 0.61 | 0.56 |
| **Leishmaniasis** | 0.0015 | 0.0011 | 27.12 | 0.47 | 0.0013 | 0.0016 | -13.96 | 0.63 | 0.38 |

**Note:** Changes = (x1-x2)/x2 × 100%; x1: average monthly incidence (observed) in 2020; x2: average monthly incidence (predicted) in 2020; the p-value was computed through two proportional tests, the p-value for emergency vs. routine was computed through a two-ratio Z test.

**Table S9. Changes in the yearly mortality rates of five vector-borne diseases in 2020 between observed and predicted values**

| **Diseases** | **Mortality rates** | | **Deaths** | | **Changes (%)** | **P value** |
| --- | --- | --- | --- | --- | --- | --- |
|  | **Observed** | **Predicted** | **Observed** | **Predicted** |  |  |
| **Total** | 0.0014 | 0.0037 | 20 | 51 | -61.89 | <0.001 |
| **EEB** | 0.0009 | 0.0029 | 12 | 41 | -69.12 | <0.001 |
| **Typhus** | 0.0000 | 0.0000 | 0 | 0 | NA | NA |
| **Dengue** | 0.0000 | 0.0001 | 0 | 1 | -100.00 | 0.23 |
| **Malaria** | 0.0005 | 0.0009 | 7 | 13 | -46.08 | 0.17 |
| **Leishmaniasis** | 0.0001 | 0.0000 | 1 | 0 | NA | NA |

**Note:** Changes = (x1-x2)/x2 × 100%; x1: yearly mortality rate (observed) in 2020; x2: yearly mortality rate (predicted) in 2020; the p-value was computed through two proportional tests.

**Table S10. Changes in the average monthly mortality rates of five vector-borne diseases in the emergency response stage (January to April 2020) and the routine response stage (May to December 2020) between observed and predicted values**

| **Diseases** | **Emergency stage (January to April)** | | | | **Routine stage (May to December)** | | | | **E vs R P-value** |
| --- | --- | --- | --- | --- | --- | --- | --- | --- | --- |
|  | **Observed** | **Predicted** | **Changes (%)** | **P value** | **Observed** | **Predicted** | **Changes (%)** | **P value** |  |
| **Total** | 0.0001 | 0.0001 | -2.23 | 0.98 | 0.0001 | 0.0004 | -72.17 | 0.14 | 0.52 |
| **EEB** | 0.0001 | 0.0000 | 19.74 | 0.91 | 0.0001 | 0.0003 | -76.44 | 0.13 | 0.62 |
| **Typhus** | 0.0000 | 0.0000 | -100.00 | 1.00 | 0.0000 | 0.0000 | -100.00 | 0.82 | 1.00 |
| **Dengue** | 0.0000 | 0.0000 | -100.00 | 0.81 | 0.0000 | 0.0000 | -100.00 | 0.69 | 1.00 |
| **Malaria** | 0.0001 | 0.0001 | -2.97 | 0.98 | 0.0000 | 0.0001 | -74.35 | 0.51 | 0.67 |
| **Leishmaniasis** | 0.0000 | 0.0000 | -100.00 | 0.82 | 0.0000 | 0.0000 | NA | NA | NA |

**Note:** Changes = (x1-x2)/x2 × 100%; x1: average monthly mortality rate (observed) in 2020; x2: average monthly mortality rate (predicted) in 2020; the p-value was computed through two proportional tests, the p-value for emergency vs. routine was computed through a two-ratio Z test.
